# Supplementary material for: In vivo rendezvous of small nucleic acid drugs with charge-matched block catiomers to target cancers
Source: Nat Commun. 2019 Apr 24;10:1894. doi: 10.1038/s41467-019-09856-w (PMC6482185; doi:10.1038/s41467-019-09856-w)
Supplement: Supplementary file 1 — Supplementary Information [file 41467_2019_9856_MOESM1_ESM.pdf]

Supplementary Information

In Vivo Rendezvous of Small Nucleic Acid Drugs with Charge-Matched Block Catiomers to Target Cancers

Sumiyo Watanabe[1,2,3], Kotaro Hayashi[4], Kazuko Toh[4], Hyun Jin Kim[1], Xueying Liu[4], Hiroyuki Chaya[1,5], Shigeto Fukushima[4], Keisuke Katsushima[6], Yutaka Kondo[6], Satoshi Uchida[7], Satomi Ogura[4,5], Takahiro Nomoto[8], Hiroyasu Takemoto[8], Horacio Cabral[7], Hiroaki Kinoh[4], Hiroyoshi Y. Tanaka[9], Mitsunobu R. Kano[9,10], Yu Matsumoto[1], Hiroshi Fukuhara[11], Shunya Uchida[2], Masaomi Nangaku[3], Kensuke Osada[7], Nobuhiro Nishiyama[8], Kanjiro Miyata[1,5], and Kazunori Kataoka[4,12]

1. Center for Disease Biology and Integrative Medicine, Graduate School of Medicine, The University of Tokyo, 7-3-1 Hongo, Bunkyo-ku, Tokyo 113-0033, Japan.
2. Division of Internal Medicine, School of Medicine, Teikyo University, 2-11-1 Kaga, Itabashi-ku, Tokyo 173-8606, Japan.
3. Division of Nephrology and Endocrinology, Graduate School of Medicine, The University of Tokyo, 7-3-1 Hongo, Bunkyo-ku, Tokyo 113-8544, Japan.
4. Innovation Center of NanoMedicine, Kawasaki Institute of Industrial Promotion, 3-25-14 Tonomachi, Kawasaki-ku, Kawasaki 210-0821, Japan.
5. Department of Materials Engineering, Graduate School of Engineering, The University of Tokyo, 7-3-1 Hongo, Bunkyo-ku, Tokyo 113-8656, Japan.
6. Division of Cancer Biology, Nagoya University Graduate School of Medicine, 65 Tsurumai-cho, Showa-ku, Nagoya 466-8550, Japan
7. Department of Bioengineering, Graduate School of Engineering, The University of Tokyo, 7-3-1 Hongo, Bunkyo-ku, Tokyo 113-8656, Japan.
8. Laboratory for Chemistry and Life Science, Institute of Innovative Research, Tokyo Institute of Technology, R1-11, 4259 Nagatsuta, Midori-ku, Yokohama 226-8503, Japan.
9. Department of Pharmaceutical Biomedicine, Okayama University Graduate School of Medicine, Dentistry, and Pharmaceutical Sciences, 1-1-1 Tsushima-naka, Kita-ku, Okayama-shi, Okayama Prefecture 700-8530, Japan.
10. Department of Pharmaceutical Biomedicine, Okayama University Graduate School of Interdisciplinary Science and Engineering in Health Systems, 1-1-1 Tsushima-naka, Kita-ku, Okayama-shi, Okayama Prefecture 700-8530, Japan.
11. Department of Urology, Graduate School of Medicine, The University of Tokyo, 7-3-1 Hongo, Bunkyo-ku, Tokyo 113-8544, Japan.
12. Policy Alternatives Research Institute, The University of Tokyo, 7-3-1 Hongo, Bunkyo-ku, Tokyo 113-0033, Japan.

S. Watanabe, K. Hayashi, K. Toh, and H. J. Kim contributed equally. K. Miyata and K. Kataoka jointly supervised this work. Correspondence and requests for materials should be addressed to K.M. (miyata@bmw.t.u-tokyo.ac.jp) and K.Kata. (kataoka@pari.u-tokyo.ac.jp).

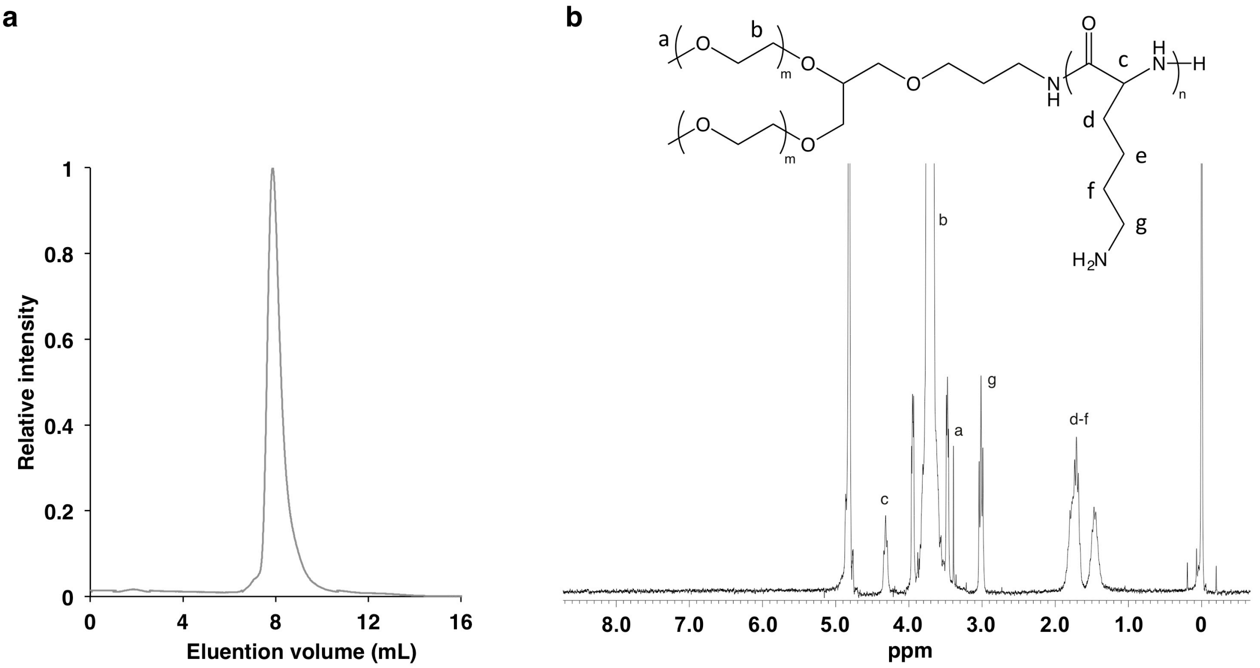

Supplementary Figure 1 | Characterisation of YBC. a, SEC chart of YBC. **b,** ^{1}H-NMR spectrum of YBC (polymer concentration: 10 mg mL^{-1}, solvent: D_2O, temperature: 20 ºC).

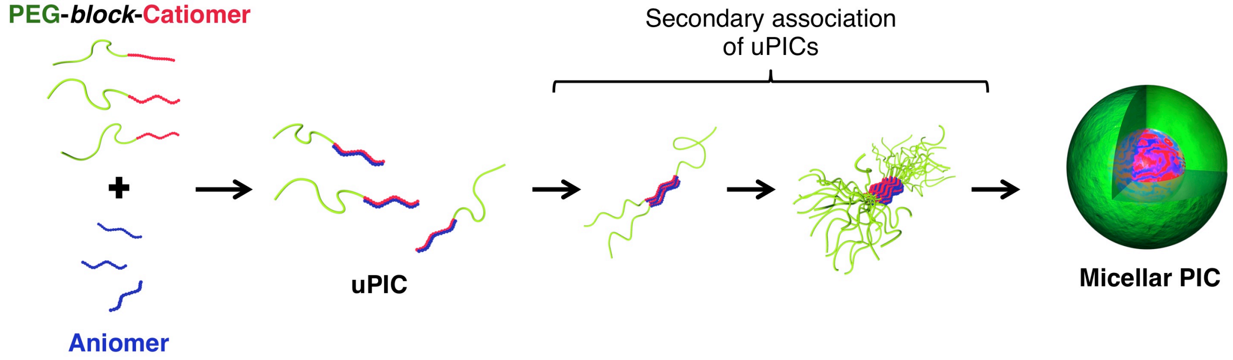

Supplementary Figure 2 | Schematic illustration of multimolecular assemblies from PEG-block-catiomers and aniomers. Micellar PICs are formed from a pair of oppositely charged block-ionomers and homo-ionomers. Charge-neutralised minimal pairs, termed uPICs, are assumed to form in the initial stage of association, followed by the secondary process of multimolecular assembly.

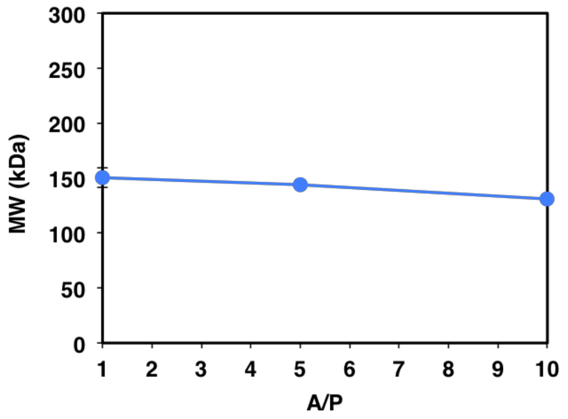

Supplementary Figure 3 | MW of uPICs prepared at varying A/P ratios. The MW was determined by SE-AUC. The uPIC (600 nM siRNA) was ultracentrifuged for over two days until an equilibrium was reached between sedimentation and diffusion, and the siRNA absorbance (260 nm) was measured as a function of centrifugal radii. The data obtained were analysed to determine the apparent MW of uPIC. Data represent the means ± s.d. for 3 replicate measurements.

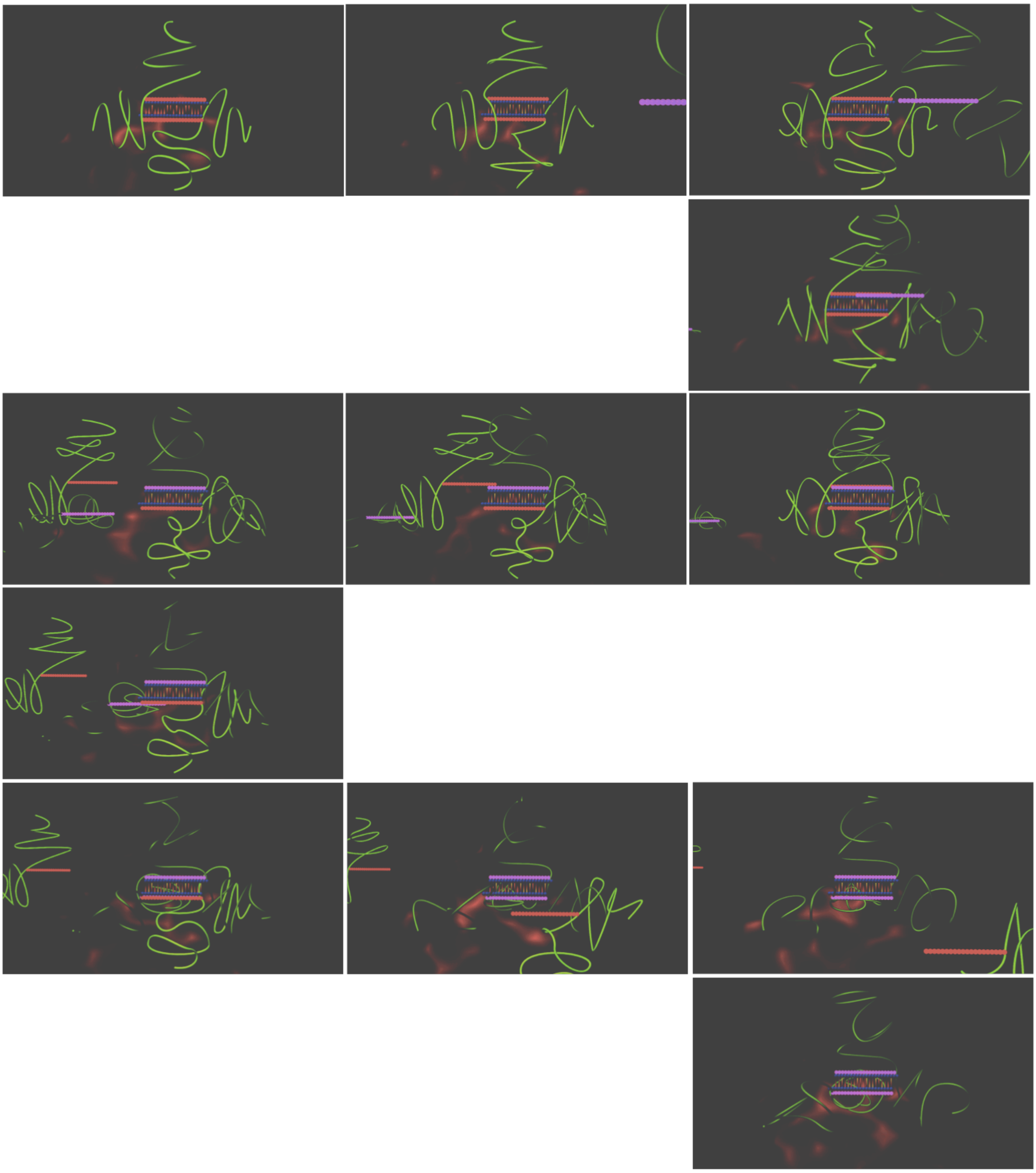

Supplementary Figure 4 | Dynamic equilibrium of uPIC with free YBC (divided images of Supplementary Movie 1).

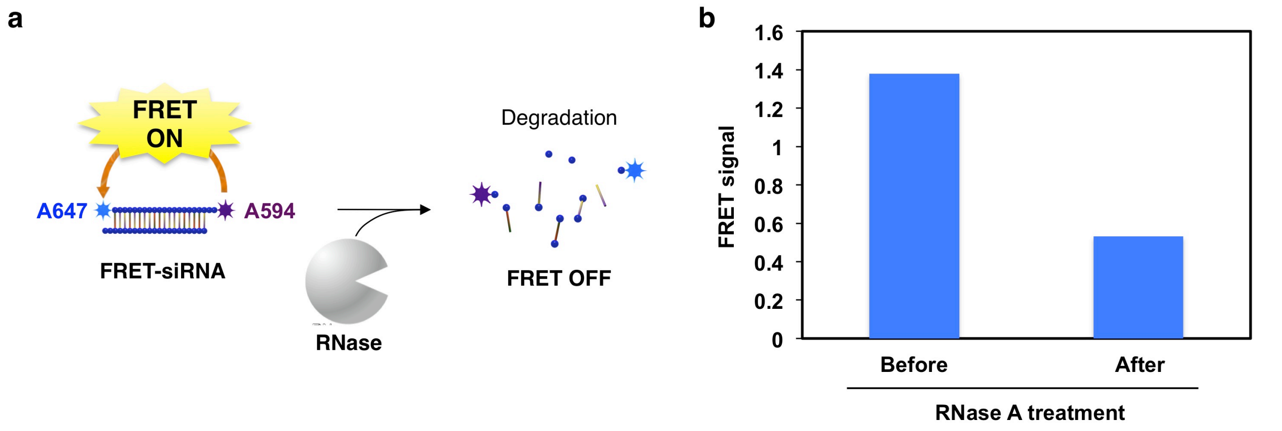

Supplementary Figure 5 | FRET analysis of siRNA integrity. a, Schematic illustration of FRET analysis of siRNA integrity. Intact FRET-siRNA generates significant FRET signal through the proximate dyes, whereas siRNA degradation elicits the loss of FRET signal due to the dye separation. **b,** FRET signal of FRET-siRNA before and after treatment with RNase A (1 μg mL^{-1}, 37 °C, 9 h). The FRET signal was defined as a fluorescence intensity ratio: [intensity at 669 nm]/[intensity at 622 nm] under excitation at 561 nm.

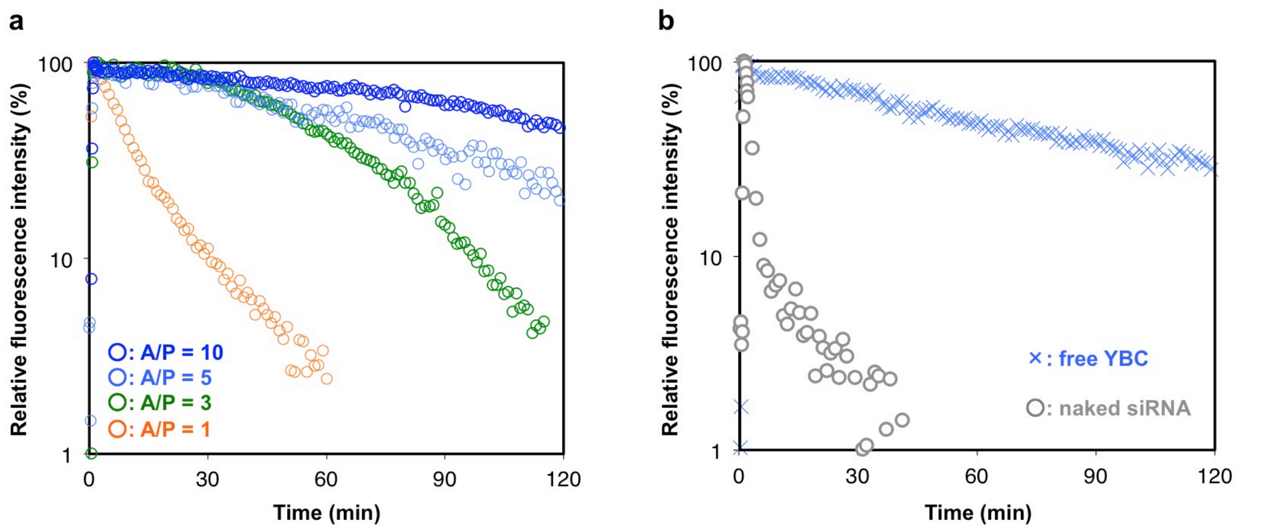

Supplementary Figure 6 | Effect of free YBC on the blood circulation property of A647-siRNA/uPICs. a, Blood circulation properties of a series of uPICs prepared at varying A/P ratios were evaluated based on the fluorescence of A647-siRNA using an IVCLSM. Each A647-siRNA/uPIC was intravenously injected from the tail vein of mouse at 2 nmol A647-siRNA per mouse, similar to Figure 3e. **b,** Blood circulation property of naked A647-siRNA (2 nmol per mouse) and free A647-YBC (40 nmol per mouse) after systemic administration into the tail vein of mouse. These dosages correspond to those of uPIC prepared at A/P = 10.

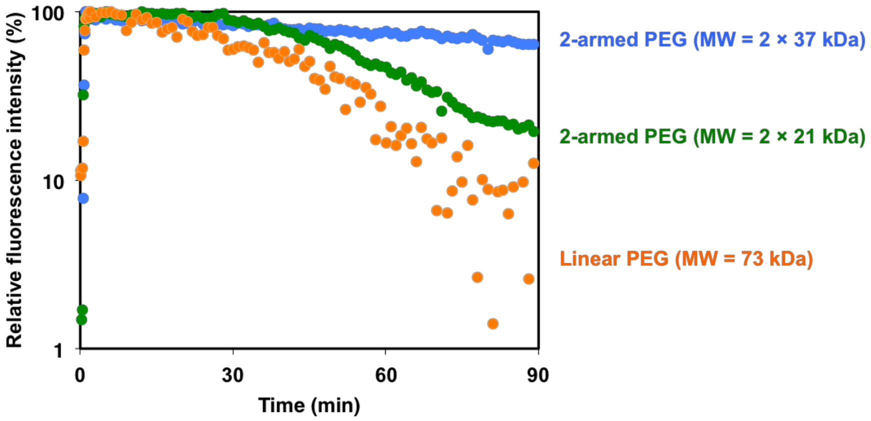

Supplementary Figure 7 | Blood circulation of A647-siRNA/uPIC comprising varying PEG chains. The blood circulation property of a series of uPICs was evaluated based on the fluorescence of A647-siRNA using an IVCLSM. Each A647-siRNA/uPIC was intravenously injected from the tail vein of mouse at 24 μg A647-siRNA per mouse, similar to Figure 3e. Control block catiomers comprising two-armed PEG with MW = 2 × 21 kDa or linear PEG with MW = 73 kDa were synthesised through a synthetic route similar to YBC with MW = 2 × 37 kDa using the corresponding PEG molecule as a macroinitiator.

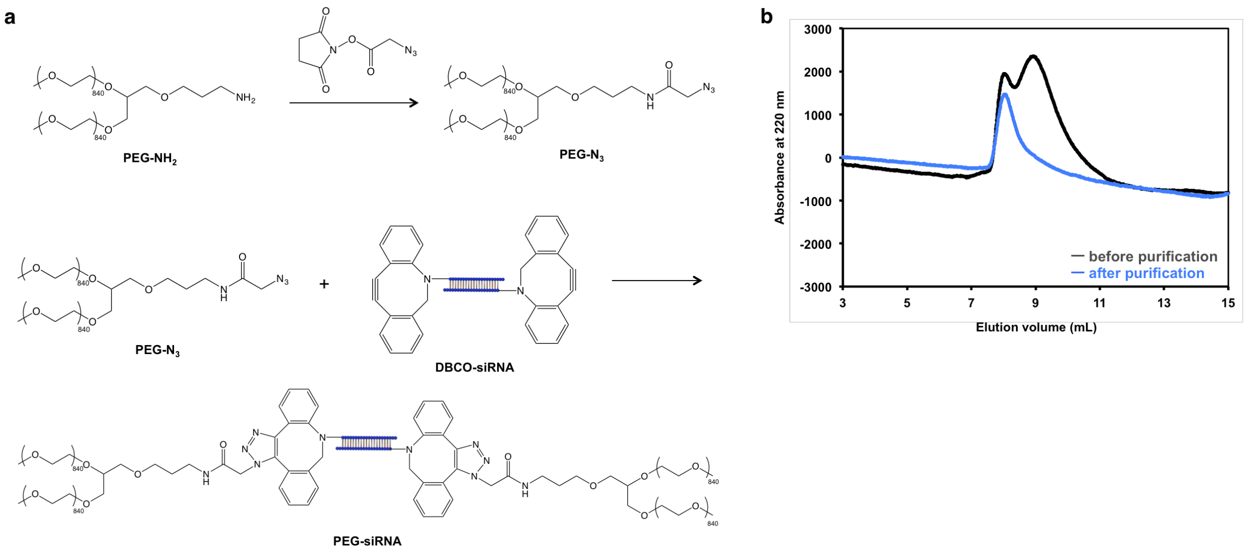

Supplementary Figure 8 | Synthesis of PEG-siRNA. a, α-Methoxy-ω-amino-two-armed PEG ($PEG\text{-}NH_2$, MW = 2 × 37 kDa) (190 mg, NOF) and azido succinimidyl acetic acid NHS (5.3 mg, Nanocs, New York, NY, USA) were dissolved in DMF and stirred for 4 h at 45 °C under Ar. *N,N*-Diisopropylethylamine (1.7 mg, Sigma-Aldrich) was then added to the mixture and further stirred overnight. The mixture was precipitated in diethyl ether, and α-methoxy-ω-azide-two-armed PEG ($PEG\text{-}N_3$) was obtained as a white powder. siRNA modified with a dibenzylcyclooctyne moiety at both 5′ ends (DBCO-siRNA) was purchased from Hokkaido System Science. DBCO-siRNA (50 μM, 100 μL in deionised water) was mixed with $PEG\text{-}N_3$ dissolved in deionised water (36 mg, 900 μL) and stirred at 40 °C for 2 days. The conjugate between DBCO-siRNA and $PEG\text{-}N_3$ was purified using AKTA explorer 10S equipped with a Superdex 200 10/300 column (GE Healthcare) eluted with 10 mM sodium phosphate buffer (pH 7.4) containing 150 mM NaCl. The product was desalted and then lyophilised. **b,** SEC charts of PEG-siRNA before and after purification. The sample was eluted in a Superdex 200 column (flow rate 0.5 mL min^{-1}, 10 mM sodium phosphate containing 150 mM NaCl, pH 7.4). The PEG-siRNA was collected as a fraction between 8 mL and 8.5 mL in the elution volume.

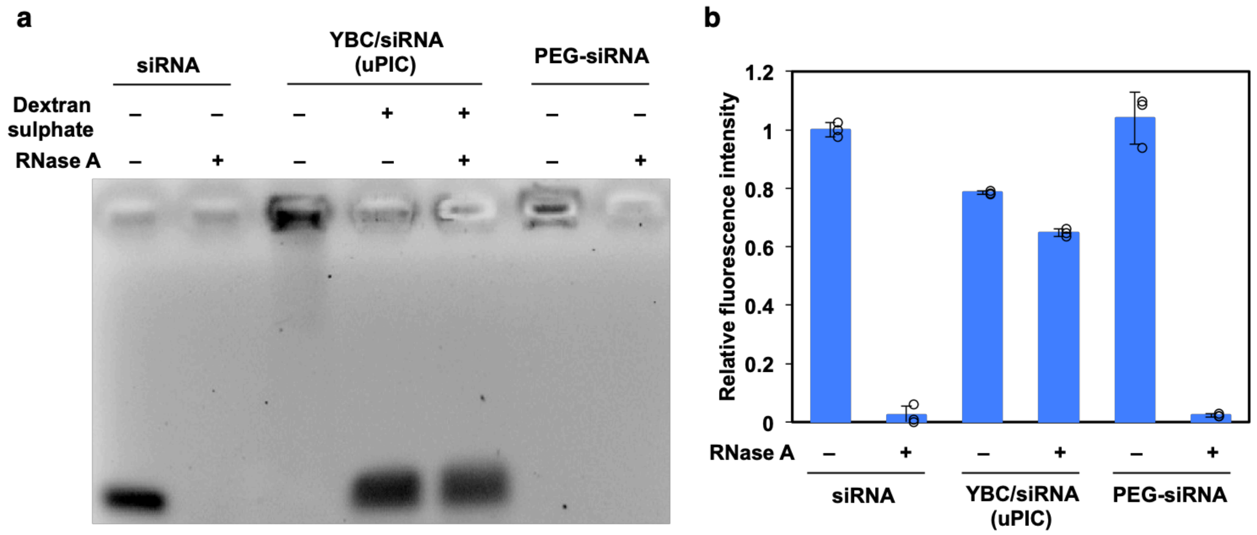

Supplementary Figure 9 | Resistance of uPIC to RNase attack. Naked siRNA, uPIC (A/P = 1), and PEG-siRNA (5 µM, 2 µL) were independently treated with RNase A (1 µM, 1 µL) for 30 min at ambient temperature. Each sample was further treated with an RNase inhibitor (4 µL, Nacalai Tesque) for 5 min, followed by a 5-min incubation with sodium dextran sulphate (50 mg mL^{-1}, 3 µL) for siRNA release from uPIC. The sample solutions were analysed by electrophoresis on 1% agarose gel with ethidium bromide visually using Molecular Imager FX (Bio-Rad) (**a**) and by fluorescence of SYBR Green I (Lonza) quantitatively using a fluorospectrometer, NanoDrop 3300 (**b**). The fluorescence intensity was normalised to that obtained from non-treated control samples. Data represent the means ± s.d. n = 3.

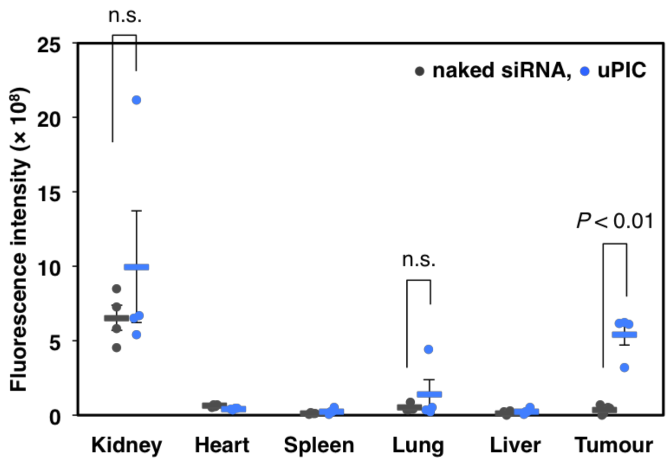

Supplementary Figure 10 | Biodistribution of naked Cy5-siRNA and Cy5-siRNA/uPIC after systemic administration. At 48 h after systemic administration (72 µg Cy5-siRNA per mouse), fluorescence intensities from the excised organs were determined using an IVIS instrument. Data represent the means (bars) ± s.e.m. with individual data points. n = 4. n.s.: not significant (P > 0.05).

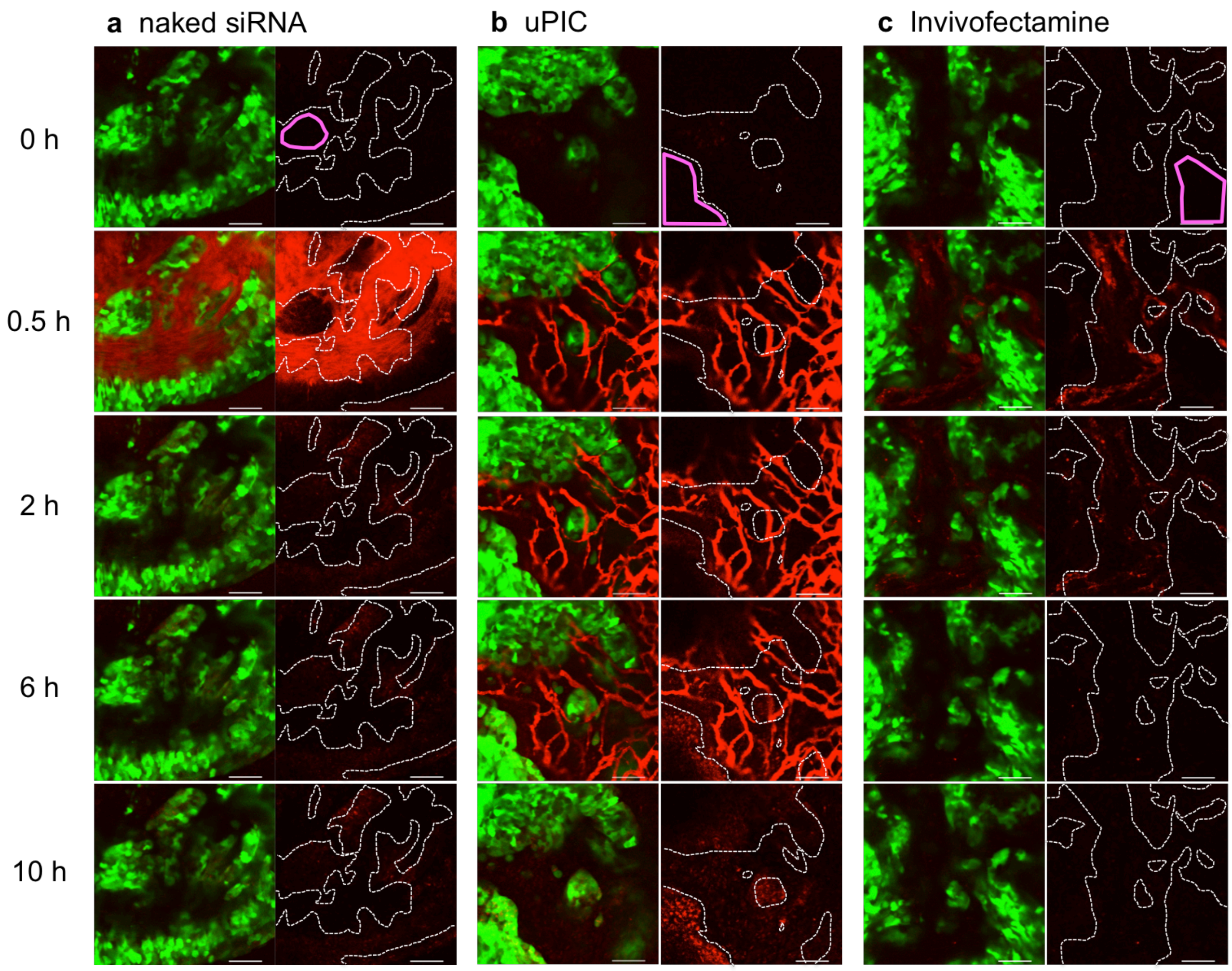

Supplementary Figure 11 | Distribution profiles of naked Cy5-siRNA, Cy5-siRNA/uPIC, and Cy5-siRNA/LNP in tumour tissue. IVCLSM images of subcutaneous GFP-BxPC3 tumour tissues after intravenous injection of Cy5-siRNA in the forms of naked (**a**), uPIC (**b**), and Invivofectamine® (**c**) (24 µg siRNA per mouse, scale bar: 100 µm). GFP and Cy5 signals are shown in green and red, respectively. Cancer cell nests are indicated by a dashed white line, as defined by the GFP signal derived from GFP-BxPC3 cells. Regions enclosed by the magenta solid line in the upper-most images (0 h) indicate the ROI used for the quantitative analysis of time-dependent changes in Cy5 fluorescence in the cancer cell nest (Fig. 3h).

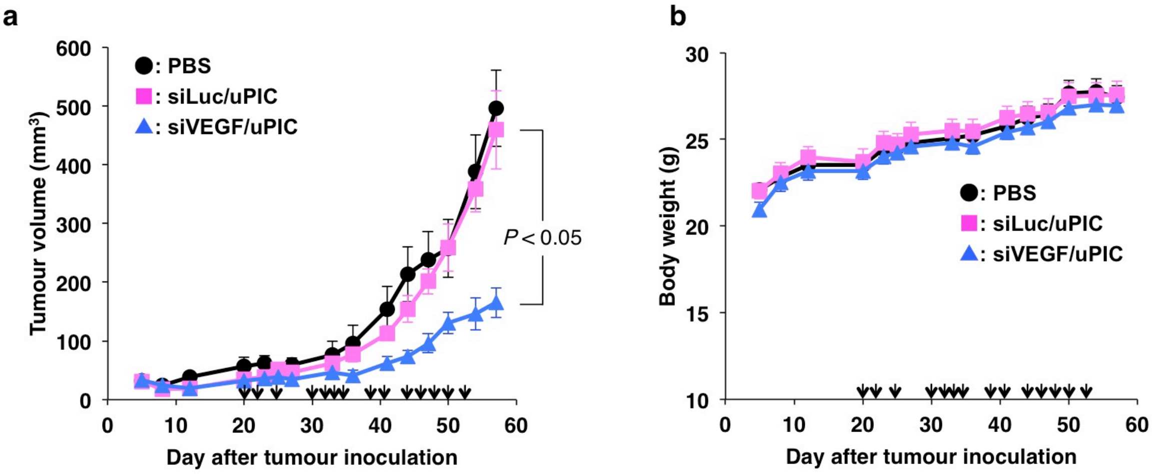

Supplementary Figure 12 | siVEGF/uPIC treatment of a subcutaneous BxPC3 tumour model. **a,** Antitumour activity of siVEGF/uPIC against subcutaneous BxPC3 tumours. siVEGF or siLuc/uPIC was intravenously injected on each measurement day into tumour-bearing mice, as indicated by arrows. Tumour size was determined by the following equation: (length of major axis) × (length of minor axis) × (height) × π/6. Data represent the means ± s.e.m. $n = 7$. **b,** Body weights of tumour-bearing mice treated with siVEGF or siLuc/uPIC described in (**a**). Data represent the means ± s.e.m. $n = 7$.

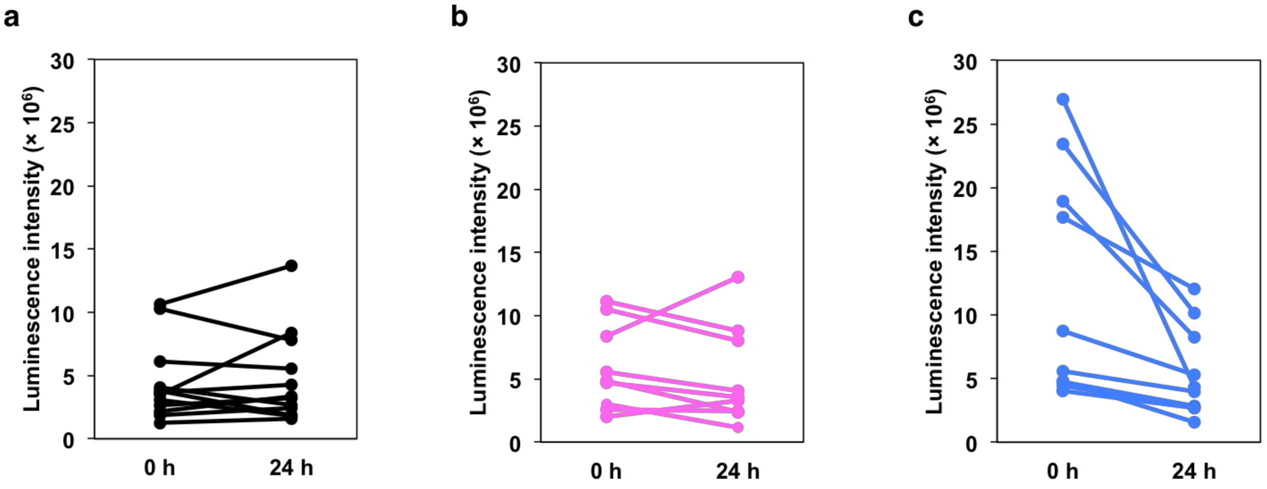

Supplementary Figure 13 | Luminescence intensity in a spontaneous pancreatic tumour model. The luminescence intensity from luciferase-expressing pancreatic tumours was measured using an IVIS instrument before and 24 h after systemic injection of PBS (**a**), siScr/uPIC (**b**), or siLuc/uPIC (**c**). uPIC solutions were prepared at A/P = 10 and the dosage of siRNA was 1.2 mg kg^{-1}.

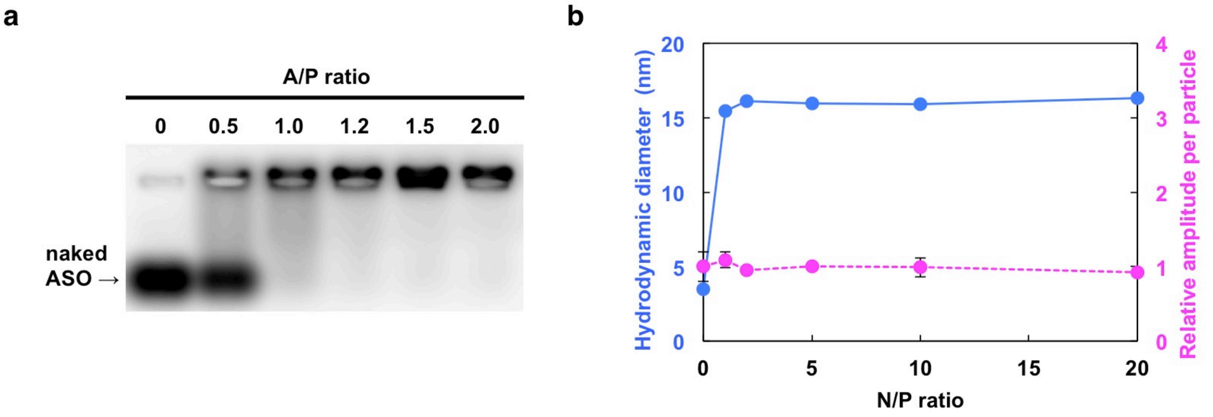

Supplementary Figure 14 | uPIC formation between ASO and YBC. a, Agarose gel retardation analysis of PIC samples prepared with ASO at varying A/P ratios. **b,** Hydrodynamic diameter of A647-ASO complexed with YBC at varying A/P ratios, and the corresponding association number of ASO per PIC, as determined by FCS. Data represent the means ± s.d. for 5 replicate measurements.

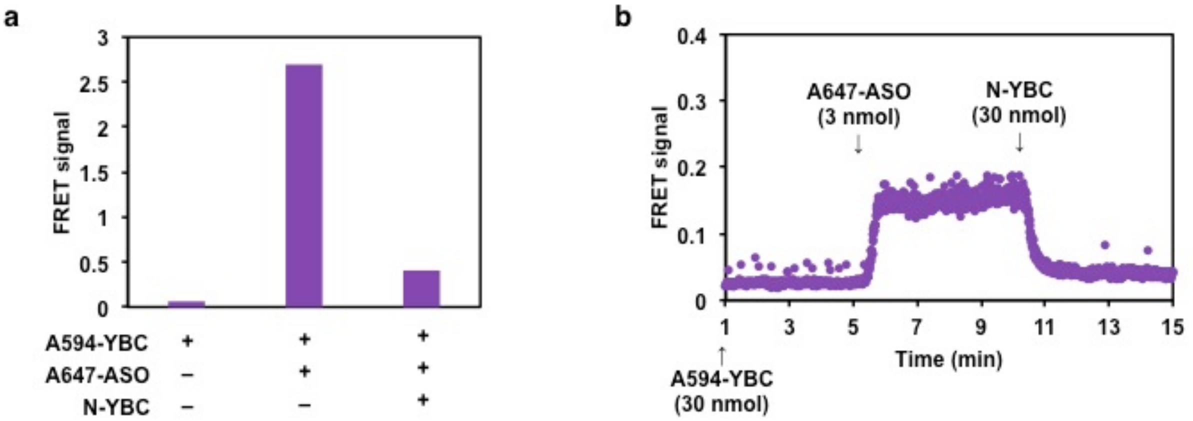

Supplementary Figure 15 | FRET analysis of the dynamic ion-pairing of ASO/uPIC with free YBC. a, Change in FRET signal of A594-YBC in buffer solution. **b,** Time-dependent change in FRET signal obtained from IVCLSM observation of the bloodstream injected sequentially with A594-YBC, A647-ASO, and N-YBC.

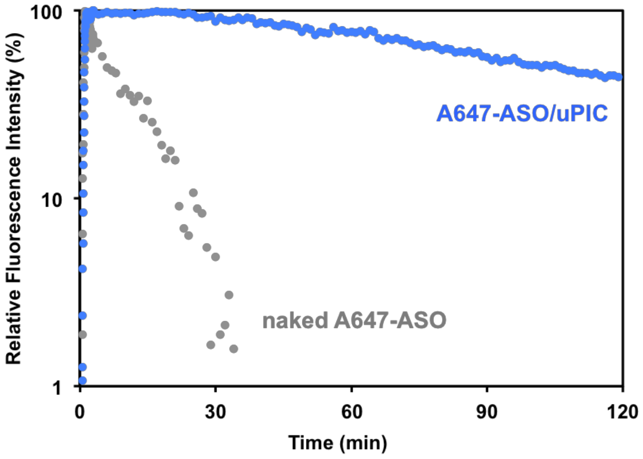

Supplementary Figure 16 | Blood circulation properties of ASO/uPIC. Time-dependent change in A647 fluorescence determined by quantitative analysis of IVCLSM data.

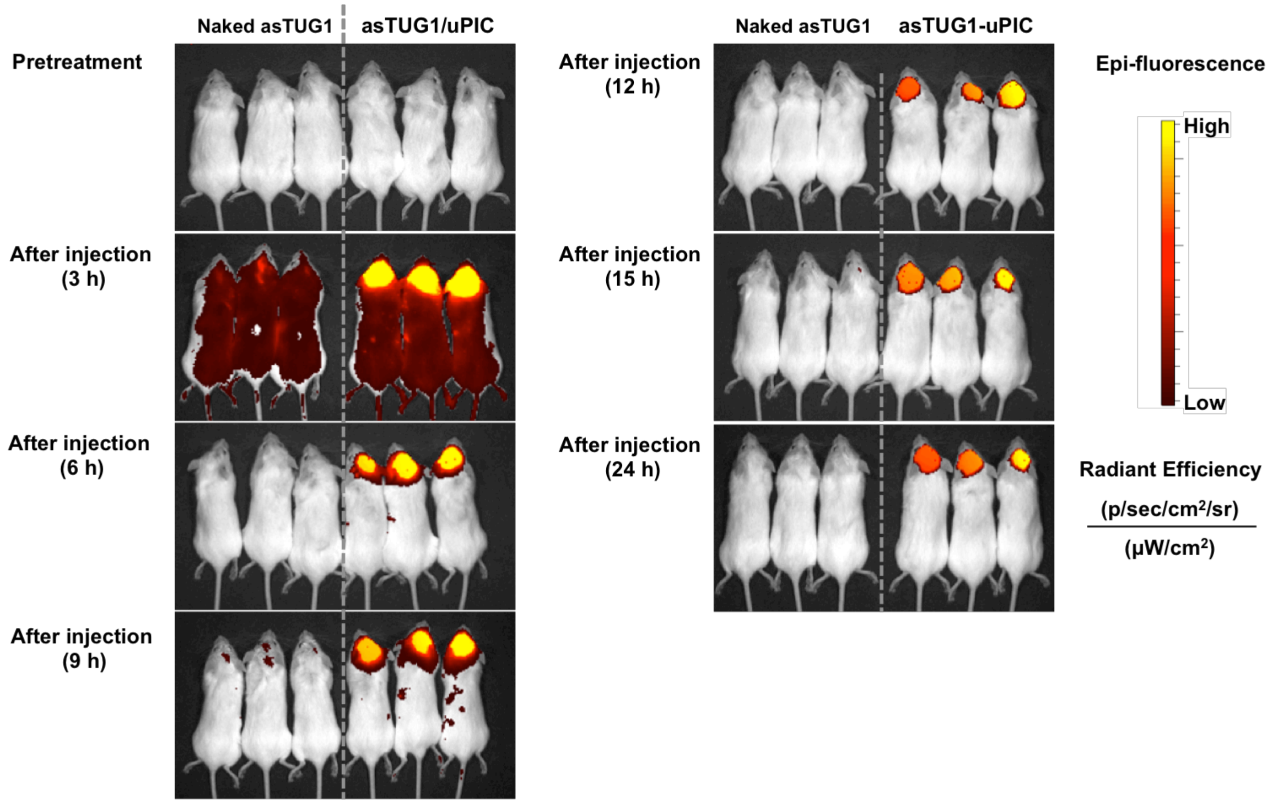

Supplementary Figure 17 | Accumulation profiles of naked ASO and ASO/uPIC in orthotopic brain tumours. Time-dependent change in A647 fluorescence in tumour-bearing mice was monitored using an IVIS instrument. These images were used for the quantitative analysis of ASO accumulation in brain tumours.

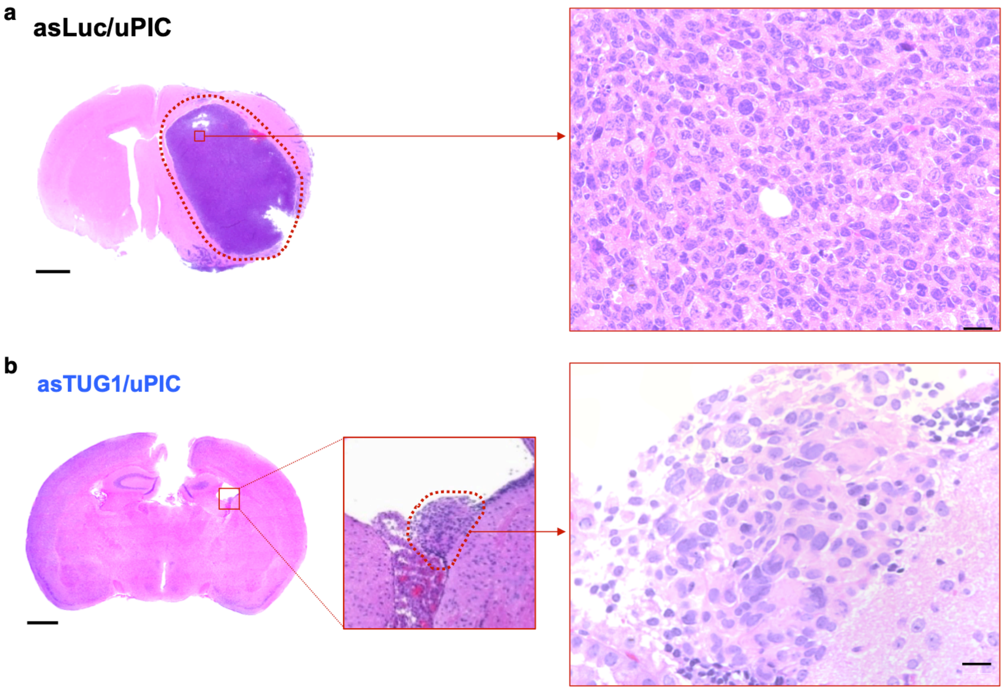

Supplementary Figure 18 | Magnified images of representative HE-stained whole brain sections shown in Figure 5c. The tumour areas are surrounded by the red dotted line. The scale bars indicate 1 mm and 20 μm in the left whole brain images and the right magnified images, respectively. The brain was harvested from the mouse treated with asLuc/uPIC (**a**) or asTUG1/uPIC (**b**).

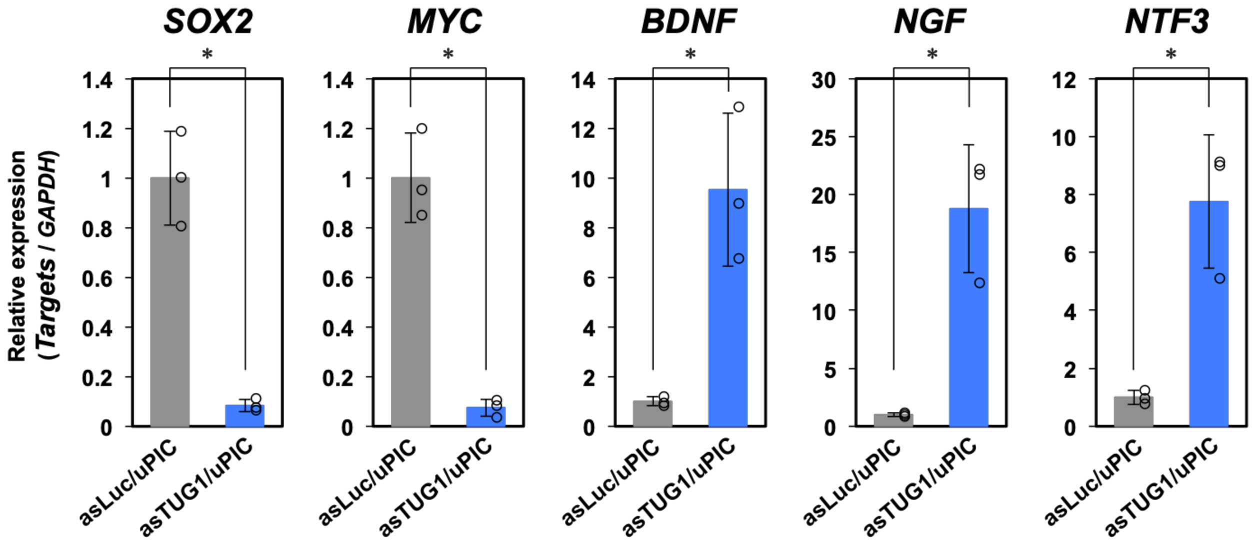

Supplementary Figure 19 | Expression profiles of stemness-associated genes (*SOX2* and *MYC*) and neuronal differentiation-associated genes (*BDNF*, *NGF*, and *NTF3*) in orthotopic brain tumour tissues after treatment with asLuc/uPIC or asTUG1/uPIC. Data represent the means ± s.d. $n = 3$. $^{*}P < 0.01$.

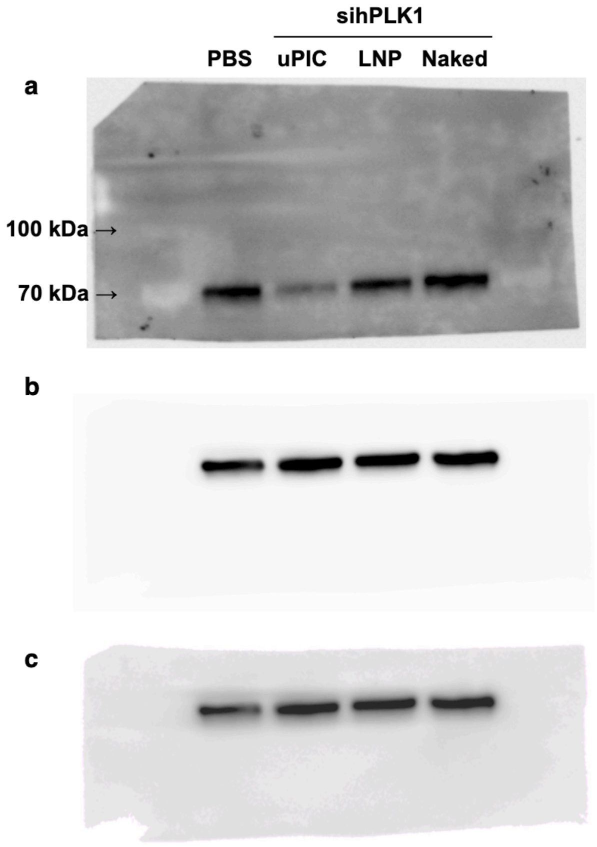

Supplementary Figure 20 | Uncropped images of western blotting gels. a,b, Unprocessed images of western blotting gels stained for *PLK1* protein (**a**) and *β-actin* protein (**b**) in the subcutaneous pancreatic tumour tissues. **c,** Slightly processed image of western blotting gel stained for *β-actin* protein.

Supplementary Table 1. Structural information of siRNA and ASO used in this study

Type	Name[a]	Sense Sequence (5' to 3')[b]	Antisense Sequence (5' to 3')[b]	Ref
siRNA	siLuc [c]	CUU ACG CUG AGU ACU UCG Att	UCG AAG UAC UCA GCG UAA Gtt	1
	sihPLK1	AGA U_MCA CCC U_MCC UU_MA AAU_M AUU	UAU UUA AG_MG AGG GUG AU_MC UUU	2
	simPLK1	GCA GCA GGA AAC CUC UCA Att	UUG AGA GGU UUC CUG CUG Ctt	3
	siVEGF	GGA GUA CCC UGA UGA GAU Ctt	GAU CUC AUC AGG GUA CUC Ctt	4
	siScr	UUC UCC GAA CGU GUC ACG Utt	ACG UGA CAC GUU CGG AGA Att	–
ASO	asLuc [c]	–	**T*C*G*A***a*g*t*a*c*t*c*a*g*c*g*t*a*a***G*T*T**	1
	asTUG1	–	**T*G*A*A***t*t*t*c*a*a*t*c*a*t*t*t*g*a***G*A*T**	5

[a] Luc: luciferase, h/mPLK1: human/mouse polo-like kinase 1, VEGF: vascular endothelial growth factor, Scr: scramble, TUG1: taurine upregulated gene 1.

[b] Uppercase: RNA, Lowercase: DNA, X_M: 2'-O-methyl-modified RNA, Bold uppercase: LNA, asterisk: phosphorothiolate.

[c] siLuc and asLuc were used as fluorescently labelled nucleic acids. Cy5- and A647-siRNA: Cy5 and Alexa Fluor 647 dyes were attached to the 5' end of the sense strand of siLuc. FRET-siRNA: Alexa Fluor 594 and 647 dyes were attached to the 3' and 5' ends of the antisense strand of siLuc. A647-ASO: Alexa Fluor 647 dye was attached to the 5' end of asLuc.

Supplementary Table 2. List of primers used in this study

Target gene	Assay ID in TaqMan™ Gene expression Assay	Primer sequence (5' to 3')	
		Forward	Reverse
hPLK1	Hs00153444_m1		
18S rRNA	Hs99999901_s1		
TUG1		AGGTAGAACCTCTATGCATTTTGTG	ACTCTTGCTTCACTACTTCATCCAG
SOX2		AAGAAAGGGAGAGAAGTTTGAGCC	GGCTCCGCGAGGAAAATC
MYC		GCTGCTTAGACGCTGGATTT	CACCGAGTCGTAGTCGAGGT
BDNF		ATTTTGGGTTAGGAGAAGTCAAGTT	GTGATCACTAACATTTTCAGGTGTG
NGF		GGACCCAATAACAGTTTTACCAAG	ACATTGCTCTCTGAGTGTGGTTC
NTF3		ACAATATTTTTATGAAACGCGATGT	CCACGAGTTTATTGTTCTCTGAAGT
GAPDH		CCTCCCGCTTCGCTCTCT	GGCGACGCAAAAGAAGATG

References

1. Elbashir, S. M. *et al*. Duplexes of 21-nucleotide RNAs mediate RNA interference in cultured mammalian cells. *Nature* **411,** 494–498 (2001).
2. Judge, A. D. *et al*. Confirming the RNAi-mediated mechanism of action of siRNA-based cancer therapeutics in mice. *J. Clin. Invest.* **119,** 661–673 (2009).
3. Raab, M. et al. Toxicity modelling of Plk1-targeted therapies in genetically engineered mice and cultured primary mammalian cells. *Nature Commun.* **2,** 395 (2011).
4. Takei, Y., Kadomatsu, K., Yuzawa, Y., Matsuo, S. & Muramatsu, T. A small interfering RNA targeting vascular endothelial growth factor as cancer therapeutics. *Cancer Res.* **64,** 3365–3370 (2004).
5. Katsushima, K. *et al*. Targeting the notch-regulated non-coding RNA TUG1 for glioma treatment. *Nature Commun.* **7,** 13616 (2016).
