# Supplementary material for: In vivo rendezvous of small nucleic acid drugs with charge-matched block catiomers to target cancers
Source: Nat Commun. 2019 Apr 24;10:1894. doi: 10.1038/s41467-019-09856-w (PMC6482185; doi:10.1038/s41467-019-09856-w)
Supplement: Supplementary file 3 — Description of Additional Supplementary Files [file 41467_2019_9856_MOESM3_ESM.pdf]

Description of Additional Supplementary Files

Supplementary Movie 1. A proposed animation of the dynamic equilibrium of uPIC with free YBC.

Supplementary Movie 2. *In vivo* FRET analysis of the dynamic equilibrium of uPIC by IVCLSM. A594-YBC was intravenously injected into the mouse, followed by sequential injections of A647-siRNA and N-YBC. The FRET signal is expressed as a rainbow scale (high: magenta, low: blue). A part of this movie is used as Figure 2c–e.

Supplementary Movie 3. Blood circulation profile of naked A647-siRNA in the murine earlobe dermis observed by IVCLSM. A part of this movie is used as Figure 3a, b, and the fluorescence intensity was quantified and is presented in Figure 3e.

Supplementary Movie 4. Blood circulation profile of A647-siRNA/uPIC in the murine earlobe dermis observed by IVCLSM. A part of this movie is used as Figure 3c, d, and the fluorescence intensity was quantified and is presented in Figure 3e.

Supplementary Movie 5. *In vivo* FRET analysis of siRNA integrity in the bloodstream. FRET-siRNA (A594/A647 dual-labelled siRNA)/uPIC was intravenously injected into the mouse. The FRET signal was quantified and is presented in Figure 3f.

Supplementary Movie 6. Time-dependent distribution profile of naked Cy5-siRNA in subcutaneous GFP-BxPC3 tumour tissue. Left: merged signal, centre: GFP signal, and right: Cy5-siRNA signal. A part of this movie is used in Supplementary Figure 11a, and the results were quantitatively analysed for presentation in Figure 3h, i.

Supplementary Movie 7. Time-dependent distribution profile of Cy5-siRNA/uPIC in subcutaneous GFP-BxPC3 tumour tissue. Left: merged signal, centre: GFP signal, and right: Cy5-siRNA signal. A part of this movie is used in Supplementary Figure 11b, and the quantitatively analysed results are represented in Figure 3h, j.

Supplementary Movie 8. Time-dependent distribution profile of Cy5-siRNA/Invivofectamine in subcutaneous GFP-BxPC3 tumour tissue. Left: merged signal, centre: GFP signal, and right: Cy5-siRNA signal. A part of this movie is used in Supplementary Figure 11c, and the quantitatively analysed results are represented in Figure 3h, k.
