# Supplementary material for: In vivo rendezvous of small nucleic acid drugs with charge-matched block catiomers to target cancers
Source: Nat Commun. 2019 Apr 24;10:1894. doi: 10.1038/s41467-019-09856-w (PMC6482185; doi:10.1038/s41467-019-09856-w)
Supplement: Supplementary file 12 — Reporting Summary [file 41467_2019_9856_MOESM12_ESM.pdf]

nature research

Corresponding author(s): Kanjiro Miyata

Last updated by author(s): Mar 18, 2019

Reporting Summary

Nature Research wishes to improve the reproducibility of the work that we publish. This form provides structure for consistency and transparency in reporting. For further information on Nature Research policies, see Authors & Referees and the Editorial Policy Checklist.

Statistics

For all statistical analyses, confirm that the following items are present in the figure legend, table legend, main text, or Methods section.

n/a	Confirmed	
☐	☒	The exact sample size (n) for each experimental group/condition, given as a discrete number and unit of measurement
☐	☒	A statement on whether measurements were taken from distinct samples or whether the same sample was measured repeatedly
☒	☐	The statistical test(s) used AND whether they are one- or two-sided<br>*Only common tests should be described solely by name; describe more complex techniques in the Methods section.*
☒	☐	A description of all covariates tested
☒	☐	A description of any assumptions or corrections, such as tests of normality and adjustment for multiple comparisons
☒	☐	A full description of the statistical parameters including central tendency (e.g. means) or other basic estimates (e.g. regression coefficient) AND variation (e.g. standard deviation) or associated estimates of uncertainty (e.g. confidence intervals)
☒	☐	For null hypothesis testing, the test statistic (e.g. F, t, r) with confidence intervals, effect sizes, degrees of freedom and P value noted<br>*Give P values as exact values whenever suitable.*
☒	☐	For Bayesian analysis, information on the choice of priors and Markov chain Monte Carlo settings
☒	☐	For hierarchical and complex designs, identification of the appropriate level for tests and full reporting of outcomes
☒	☐	Estimates of effect sizes (e.g. Cohen's d, Pearson's r), indicating how they were calculated

Our web collection on statistics for biologists contains articles on many of the points above.

Software and code

Policy information about availability of computer code

Data collection	No software was used.
Data analysis	No software was used.

For manuscripts utilizing custom algorithms or software that are central to the research but not yet described in published literature, software must be made available to editors/reviewers. We strongly encourage code deposition in a community repository (e.g. GitHub). See the Nature Research guidelines for submitting code & software for further information.

Data

Policy information about availability of data

All manuscripts must include a data availability statement. This statement should provide the following information, where applicable:

- Accession codes, unique identifiers, or web links for publicly available datasets
- A list of figures that have associated raw data
- A description of any restrictions on data availability

No datasets were generated or analysed during the current study.

Field-specific reporting

Please select the one below that is the best fit for your research. If you are not sure, read the appropriate sections before making your selection.

☒ Life sciences ☐ Behavioural & social sciences ☐ Ecological, evolutionary & environmental sciences

For a reference copy of the document with all sections, see nature.com/documents/nr-reporting-summary-flat.pdf

Life sciences study design

All studies must disclose on these points even when the disclosure is negative.

Sample size	In all experiments, the sample sizes were determined by estimating the minimum number of samples to obtain a statistically significant difference.
Data exclusions	In atitumour experiments (Figure 4i and 4l), the data that were more largely deviated from the average value were excluded to make the sample size uniform.
Replication	Major experiments were performed once with an appropriate sample size to obtain statistical significances.
Randomization	In in vivo experiments using tumor-bearing mice, mice were randomly allocated to each group to have the similar tumour size in average.
Blinding	The investigators were blinded for data collection.

Reporting for specific materials, systems and methods

We require information from authors about some types of materials, experimental systems and methods used in many studies. Here, indicate whether each material, system or method listed is relevant to your study. If you are not sure if a list item applies to your research, read the appropriate section before selecting a response.

Materials & experimental systems

n/a	Involved in the study	
☐	☒	Antibodies
☐	☒	Eukaryotic cell lines
☒	☐	Palaeontology
☐	☒	Animals and other organisms
☒	☐	Human research participants
☒	☐	Clinical data

Methods

n/a	Involved in the study	
☒	☐	ChIP-seq
☒	☐	Flow cytometry
☒	☐	MRI-based neuroimaging

Antibodies

Antibodies used	Anti-Argonaute 2 antibody: Wako Pure Chemical Industries, 015-22031<br>Anti-human PLK1 antibody: Cell Signaling Technology, #4513<br>Anti human beta-actin antibody: Cell Signaling Technology, #4967<br>Anti HRP-linked secondary antibody: Cell Signaling Technology, #7074<br>Anti-GFP rabbit monoclonal antibody: Invitrogen, A-6455<br>Alexa Fluor488-conjugted anti-rabbit IgG secondary antibody: Invitrogen, R37116
Validation	Anti-Argonaute 2 antibody: human species reactivity; western blotting(WB), immunoprecipitation (IP), immunocytochemistry (ICC) applicable.<br>Anti-human PLK1 antibody: human, rat, monkey species reactivity; WB, IP, Immunohistochemistry (IH) applicable.<br>Anti-human beta-actin antibody: human, mouse, rat, hamster, monkey, mink, D. melanogaster, zebrafish, bovin species reactivity; WB applicable.<br>Anti-GFP antibody: Tag species reactivity; ELISA, ICC, Immunofluorescence, IH applicable.

Eukaryotic cell lines

Policy information about cell lines

Cell line source(s)	BxPC3: American Type Culture Collection<br>GFP-expressing BxPC3: AntiCancer Japan<br>Glioma stem cells: Nagoya University Hospital
Authentication	GFP-expressing BxPC3 was authenticated by STR-PCR.<br>Stem cell markers in the glioma stem cells were authenticated by RT-PCR. Please see these references. (1) K. Yuki, et al, Cancer Letters, 284 (2009) 71-79. (2) K. Katsushima, et al, Nature Communications, 7 (2016) 13616.
Mycoplasma contamination	BxPC3 cells were used after confirming no mycoplasma contamination.
Commonly misidentified lines<br>(See ICLAC register)	No commonly misidentified lines were used in this study.

Animals and other organisms

Policy information about studies involving animals; ARRIVE guidelines recommended for reporting animal research

Laboratory animals	BALB/c mice (6-week-old female) were used for blood circulation test.<br>BALB/c nude mice (6-week-old male) were used for developing a subcutaneous BxPC tumour model.<br>EL1-luc-TAg FVB/N mice (13 weeks-old male) were used for developing spontaneous pancreatic cancer model.<br>NOD/SCID mice (6-week-old female) were used for developing an orthotopic brain tumor.
Wild animals	No wild animals were used in this study.
Field-collected samples	No field-collected samples were used in this study.
Ethics oversight	Animal experiments using healthy mice and subcutaneous tumour-bearing mice were performed in accordance with the guidelines for animal experiments at The University of Tokyo, Japan.<br>Experiments involving oncomice were performed in accordance with the ethics committee of the Innovation Center of NanoMedicine, Japan.<br>Experiments involving orthotopic brain tumour-bearing mice were performed in accordance with the protocols approved by the Institutional Animal Care and Use Committee of Nagoya University Graduate School of Medicine, Japan, respectively.

Note that full information on the approval of the study protocol must also be provided in the manuscript.
